# TPP1 Enhances the Therapeutic Effects of Transplanted Aged Mesenchymal Stem Cells in Infarcted Hearts via the MRE11/AKT Pathway

**DOI:** 10.3389/fcell.2020.588023

**Published:** 2020-10-29

**Authors:** Kaixiang Yu, Zhiru Zeng, Si Cheng, Wangxing Hu, Chenyang Gao, Feng Liu, Jinyong Chen, Yi Qian, Dilin Xu, Jing Zhao, Xianbao Liu, Jian’an Wang

**Affiliations:** ^1^Department of Cardiology, The Second Affiliated Hospital, Zhejiang University School of Medicine, Hangzhou, China; ^2^Cardiovascular Key Laboratory of Zhejiang Province, Hangzhou, China

**Keywords:** stem cells therapy, myocardial infarction, aging, DNA repair, telomere

## Abstract

**Background:**

Poor cell survival after transplantation restricts the therapeutic potential of mesenchymal stem cell (MSC) transplantation into infarcted hearts, particularly in older individuals. TPP1, a component of the shelterin complex that is involved in telomere protection, is highly expressed in young MSCs but declines in aged ones. Here, we explore whether TPP1 overexpression in aged mouse MSCs improves cell viability *in vivo* and *in vitro*.

**Methods:**

Aged mouse MSCs overexpressing TPP1 were injected into the peri-infarct area of the mouse heart after left anterior descending coronary artery ligation. In parallel, to evaluate cellular-level effects, H_2_O_2_ was applied to MSCs *in vitro* to mimic the microenvironment of myocardial injury.

**Results:**

*In vivo*, the transplantation of aged MSCs overexpressing TPP1 resulted in improved cell survival, enhanced cardiac function, and reduced fibrosis compared to unmodified aged MSCs. *In vitro*, TPP1 overexpression protected aged MSCs from H_2_O_2_-induced apoptosis and enhanced DNA double-strand break (DSB) repair. In addition, the phosphorylation of AKT and the key DSB repair protein MRE11 were both significantly upregulated in aged MSCs that overexpressed TPP1.

**Conclusions:**

Our results reveal that TPP1 can enhance DNA repair through the AKT/MRE11 pathway, thereby improving the therapeutic effects of aged MSC transplantation and offering significant potential for the clinical application of autologous transplantation in aged patients.

## Introduction

Mesenchymal stem cells (MSCs) are a promising cell type for treating ischemic heart diseases due to their multipotency, capacity for self-renewal, and immune-privileged status (Williams et al., 2011; Broughton and Sussman, 2016; Golpanian et al., 2016). MSCs from older patients (the source of autologous stem cell therapy) have shorter telomere length than those of their young, healthy counterparts, as well as impaired stem cell properties, including proliferation, differentiation, and paracrine secretion. These observations likely explain the fact that clinical trials of autologous cell transplantation show limited therapeutic effects in the setting of myocardial infarction in older patients (Nurkovic et al., 2016). Because older patients are the target population for this therapy, novel strategies are needed to enhance the therapeutic potential of aged MSCs for autologous cell transplantation in myocardial ischemia.

The shortening of telomeres plays a crucial role in the cellular aging process (Shay, 2018). Aging is accompanied by increased DNA damage and diminished DNA repair capacity, which is counteracted in part by telomeres. Human telomeres are DNA sequences at the end of chromosomes, capped by six kinds of associated proteins called shelterin, which protect the telomeric DNA from being recognized as double-strand DNA breaks and leading to end-to-end fusions (Cosme-Blanco and Chang, 2008; Palm and de Lange, 2008; de Lange, 2009). The shelterin complex is composed of six core proteins: RAP1, TRF1, TRF2, TPP1, POT1, and TIN2 (Palm and de Lange, 2008). Among these, TPP1 actively recruits telomerase to telomeres, and together with POT1, it protects chromosome ends and regulates telomere length (Kibe et al., 2010; Rajavel et al., 2014). Defects in its protection lead to the initiation of DNA damage and cell apoptosis (Guo et al., 2007). Telomere length decreases with aging, and some premature aging syndromes have been linked to shelterin protein mutations, including in TIN2, TRF1, and TRF2 (Carroll and Ly, 2009). However, it is unknown whether TPP1 plays a part in DNA damage and repair in aged MSCs or whether targeting damage and repair impacts the therapeutic efficacy of these cells.

Herein, we demonstrate that augmenting TPP1 can enhance the therapeutic efficacy of aged MSCs in myocardial infarction. The underlying mechanisms in this process involve enhanced DNA repair. These findings provide new insights into strategies to deploy autologous stem cell transplantation therapy in aged patients.

## Materials and Methods

### Cell Culture

hMSCs were isolated from patients undergoing hip replacement surgery (with young donors aged below 40 years and aged donors aged above 65 years). Informed consent was collected from each donor. The study protocol was approved by the Ethics Committee of the Second Affiliated Hospital of Zhejiang University. Cells were cultured in Dulbecco’s modified Eagle’s medium (DMEM) (GIBCO, Life Technologies, United States) with 10% fetal bovine serum (FBS, Gibico, United States) added. The culture was maintained in a humidified atmosphere containing 95% air/5% CO_2_ at 37°C. Using 0.25% trypsin with 0.2% EDTA, cells were passaged at a ratio of 1:2 when they reached 70–80% confluence. Passage was performed with 4–6 cells in this study.

### Cell Identification

The antigen profile of the MSCs was determined by flow cytometry. Passages 4–6 of hMSCs were stained at room temperature for 1 h with surface molecular-specific antibodies (FITC-CD45 for hematopoietic surface marker; PE-CD29, APC-CD90, PE-CD44 for mesenchymal surface markers; and FITC-CD34 for endothelial cell surface marker and isotype-matched control). The expression of the cell surface markers was quantified with flow cytometry (BD Biosciences, CA, United States).

Adipogenic, chondrogenic, and osteogenic differentiation of hMSCs was conducted as previously reported (Hu et al., 2016). hMSCs were seeded at density of 1.5–2 × 10^5^ per well in a six-well plate overnight to allow attachment. For osteogenesis, hMSCs were incubated with osteogenic medium for 3 weeks. Differentiation was evaluated by Alizarin-Red Staining. For chondrogenesis, hMSCs were treated with chondrogenic medium for 3 weeks and assessed with toluidine blue staining. Adipogenic differentiation was induced with adipogenesis induction medium for 23 days. Oil Red O solution was used to visualize the fat vacuoles. Images were obtained from a light microscope.

Most of the MSCs were positive for CD90 (97.3%), CD29 (99.6%), and CD44 (99.7%) expression, and negative for the hematopoietic markers CD34 (0.2%) and CD45 (0.5%) (Supplementary Figures 1A–E). The multiple differentiation potential of adipogenesis, chondrogenesis, and osteogenesis in the MSCs were also identified (Supplementary Figures 1F–H).

### Lentivirus Infection

Recombinant lentivirus containing TPP1 tagged with a flag was constructed by Hanbio (Shanghai, China), and an empty vector served as control. Each group of lentivirus expressed green fluorescent protein (GFP). hMSCs were seeded at a density of 6 × 10^4^ in a six-well plate. After cell attachment, cells were infected with lentivirus (MOI = 20) in the presence of 8 μg/mL polybrene (Sigma) overnight. Then, the medium was replaced with fresh medium, and cells were cultured for another 24 h. The transduction efficiency was evaluated by Western blotting and fluorescence microscopy.

### Small Interfering RNA Transfection

Small interfering RNA (siRNA) targeting human TPP1 (siTPP1) or MRE11 (siMRE11) and control siRNA (si-con) were obtained from Ribobio (Shanghai, China). When the MSCs reached 75% confluence, cells were transfected with siRNA using Lipofectamine RNAi MAX (Invitrogen, CA, United States) according to the manufacturer’s protocol. After 8 h incubation (37°C), the transfection mixture was replaced by fresh medium; 48 h after transfection, the cells were harvested for further experiments.

### Cell Apoptosis Model

To mimic the hypoxic aspects of the *in vivo* ischemic condition, an H_2_O_2_-induced apoptosis model was used for hMSCs. After transfection by lentivirus for 48 h, OMSC^null^ (Aged-MSCs infected with Len-null) or OMSC^TPP1^ (Aged-MSCs infected with Len-TPP1) were treated with 1,000 μM H_2_O_2_ in DMEM without FBS (5% CO_2_, at 37°C) for 2 h *in vitro*. An OGD apoptosis model was also used for prolonged cell stress. After transfection, the hMSC medium was replaced by DMEM without glucose and FBS, and the cells were cultured under hypoxic conditions (0.5% O_2_/5% CO_2_) at 37°C for 24 h.

### Tube Formation

In the normoxia group, hMSCs (2 × 10^5^ cells) transfected with lentivirus (Len-null or Len-TPP1) were incubated with DMEM and without FBS under normoxic culture. In the OGD group, cells were cultured with DMEM without glucose and FBS under hypoxic conditions. After 24 h incubation, the conditioned medium was collected from both groups. Additionally, DMEM without FBS and hMSCs incubation was also prepared to serve as the control. Human umbilical vein endothelial cells (HUVECs) transfected with GFP at 1.5 × 10^4^/well were plated in the above different conditioned medium in 96-well plates that were precoated with 50 μL Matrigel (BD, NJ, United States). After 4–8 h plantation, the tube formation of the HUVECs was captured with a fluorescence microscope and was quantified by counting the number of tubes in each well.

### Cardiomyocytes Apoptosis

The conditioned medium of OMSC^null^ or OMSC^TPP1^ after OGD and DMEM without glucose and FBS was collected, as previously described. H9C2 cardiomycytes in the 6-well plate overnight for cell adherent at a density of 2 × 10^5^. Then, the H9C2 medium was replaced with the above-conditioned medium and cultured under hypoxic conditions (0.5% O_2_/5% CO_2_) at 37°C for 24 h. Following that, the apoptosis rate of H9C2 was assessed using Annexin V-APC/PI staining and flow cytometry.

### Annexin V-APC/PI Staining

Cells were suspended in PBS and stained with a mixture of Annexin V-APC and propidium iodide (PI) dye for 30 min according to the manufacturer’s protocol of Annexin V-APC/PI Apoptosis Detection Kit (SunGENE, Tianjin, China) and detected by flow cytometry (BD Biosciences, CA, United States).

### Echocardiography

Heart function was assessed by transthoracic echocardiography before MI and at 3, 7, 14, and 28 days after it. Left ventricular (LV) M-mode and two-dimensional images were obtained, and cardiac function was analyzed with a Vevo 2100 system (Visual Sonics, Toronto, ON, Canada). The LV parameters measured included ejection fraction (EF), fractional shortening (FS), end-diastolic dimension, and end-systolic dimension. All parameters were measured in at least three separate cardiac cycles.

### Quantitative PCR

RNA was extracted from cells with the Trizol reagent (Invitrogen, Thermo Fisher Scientific). Primers were purchased from Tsingke (Shanghai, China). Quantitative PCR was performed using SYBR Green PCR Master Mix (Takara, Japan) on a 7500 Fast Real Time PCR System (Applied Biosystems, Carlsbad, CA, United States). β-actin was used as a housekeeping gene, and data were determined with the comparative ΔΔCt method. The primers are shown in Supplementary Table 1.

### Immunofluorescence Staining

Tissue slices and cells were fixed with 4% paraformaldehyde, permeabilized with 0.2% Triton-100 and blocked with 3% BSA in PBS for 1 h. Immunohistochemistry was performed with the following primary antibodies: Phospho-Histone H2A.X (Ser139) rabbit antibody (1:1000, CST, #9718), GFP primary antibody (1:400, Abcam), α-SMA (1:400, Abcam), CD31 (1:200, BD Bioscience), Cardiac Troponin I (1:200, Abcam), and anti-rabbit, anti-goat, and anti-mouse secondary antibody (1:400, CST). It was incubated with conjugated secondary antibody for 1 h at room temperature. Finally, diamidino-2-phenylindole (DAPI; blue) was used to stain the nucleus after washing twice by PBS. Fluorescent imaging was performed via a fluorescent microscope (Leica, Wetzlar, Germany).

### γ-H2AX Foci Assay

Cells were seeded into a 24-well plate and exposed to 1,000 μM H_2_O_2_ in DMEM without FBS for 1 h at 37°C. Then, they were recovered in fresh medium for 1 h. The imaging was captured by fluorescent microscope. Cells with 10 or more foci were counted as positive cells, which we interpreted as cells with unrepaired DNA double-strand breaks.

### Alkaline Comet Assay

The alkaline comet assay was performed using the Trevigen Comet Assay kit (Trevigen, Gaithersburg, MD, United States). Cells at 1 × 10^5^/mL were suspended in PBS and mixed with molten LMAgarose at a ratio of 1:10 (v/v), and 50 μL mixture was immediately pipetted onto the slide. The slide was incubated at 4°C for 10 min to accelerate gelling and was then immersed into cold lysis solution overnight at 4°C. Next, excess buffer was drained from the slides, and they were immersed into alkaline unwinding solution for 1 h at 4°C, in the dark. The slides were subjected to electrophoresis with 4°C Alkaline Electrophoresis Solution at 21 V for 30 min and immersed twice in H_2_O for 5 min each and then in 70% ethanol for 5 min, following which, they were air dried. DNA was stained with 100 μL SYBR GREEN (Invitrogen, 1:10,000) for 10 min and was immediately rinsed with H_2_O and dried. The slides were viewed with a fluorescence microscope and analyzed with Comet Score Freeware v. 1.5 (TriTek, Niigata, Japan).

### Sirius Red Staining

Then, 28 days after MI, all mice were sacrificed for histological study. The hearts were embedded into a Tissue Tek OCT compound (Sakura Finetek, Torrance, CA, United States) and cut into 7 μm thick sections. Fibrotic area was assessed using Sirius red staining kit (Solarbio Life Sciences, Beijing, China) according to the manufacturer’s instructions. The percentage of the infarct area was quantified with ImagePro-Plus software (Media Cybernetics, Rockville, MD, United States) and calculated according to the following the formula: infarct heart area (%) = (the sum of endocardial length and epicardial length of the infarcted area/the sum of endocardial and epicardial length of whole left ventricle) × 100%.

### MI Model and MSC Transplantation

All animal procedures were performed in accordance with the Guide for the Care and Use of Laboratory Animals published by the US National Institutes of Health (Publication No. 86-23, revised 1996) and were approved by the Animal Care and Use Committee of Zhejiang University. Myocardial infarction was established in male C57BL/6J mice (20–25 g, 10–12-weeks old) according to previously described methods (Hu et al., 2014). MI was induced by ligation of the left anterior descending coronary artery with a 6-0 silk suture. 10 min after the ligation, OMSC^null^ (1.5 × 10^5^ cells in 20 μL DMEM per mouse), OMSC^TPP1^ (1.5 × 10^5^ cells in 20 μL DMEM per mouse), or DMEM alone was intra-myocardially injected into the ischemic border zone of hearts at five sites. The control animals underwent MI surgery and were injected with DMEM only.

### TUNEL Staining

The apoptosis of cells was detected with an *In Situ* Cell Death Detection Kit (Roche United States). The samples were fixed in 4% paraformaldehyde, permeabilized with 0.2% TritonX-100, and incubated with deoxynucleotidyltransferase-mediated dUTP nick-end labeling (TUNEL) reaction at 37°C for 1 h in the dark. Nuclei were stained with DAPI for 10 min. The images were observed under a fluorescence microscope, and the apoptotic ratio was calculated as the number of TUNEL-positive cells/total number of cells.

### Western Blot

Proteins were separated by SDS-PAGE gel with a current of 30 mA and transferred onto PVDF membrane with a current of 300 mA. After blocking with 5% milk for 1 h, they were incubated overnight with the following primary antibodies: anti-MRE11 (1:1000, Abcam), anti-AKT (1:1000, CST); anti-pAKT (phosphor s473) (1:1000, CST), anti-PTOP (1:1000, Abcam), anti-cleaved caspase-3 (Asp175) (1:1000, CST), and anti-caspase-3 (1:1000, CST) at 4°C. Then, the membranes were washed three times with PBST and incubated with HRP-conjugated secondary antibodies (1:5000). After being washed by PBST, the protein bands were treated with ECL and detected with the Gel Doc EZ Imaging System (Bio-Rad, Berkeley, CA, United States).

β-actin was obtained from BD Biosciences, (San Jose, CA, United States); GAPDH, β-Tubulin, and HRP-conjugated anti-rabbit and anti-mouse secondary antibodies were obtained from Cell Signaling Technology.

### PI3K Inhibitor Treatment

PI3K inhibitor LY294002 was obtained from Selleckchem (Houston, TX, United States). For the pretreatment, cells were cultured with DMEM containing 10% FBS and 10 μM LY294002 under 5% CO_2_ at 37°C for 24 h. When cells were treated with H_2_O_2_, 10 μM LY294002 was also persistently contained in the DMEM with it for the PI3K inhibition group.

### Telomerase Activity Assay

Telomerase activity assay was performed with the Telo TAGGG telomerase PCR ELISA kit (Roche, Mannheim, Germany) according to the manufacturer’s instructions. Cells were harvested, centrifuged, and resuspended by 200 μL lysis reagent. The lysate was then centrifuged at 16,000 × *g* at 4°C for 20 min, and the supernatant was transferred to a fresh tube to perform the TRAP reaction. After amplification, the amplification product was added to the mixture of denaturation reagent and hybridization buffer. Next, 100 μL mixture was transferred into each well of the precoated MP modules supplied with the kit, and the MP modules were incubated at 37°C on a shaker (300 rpm) for 2 h. The hybridization solution and anti-DIG-POD working solution were added and removed, one after the other. Finally, TMB substrate solution with stop reagent was added, and a microplate (ELISA) reader was used to measure the absorbance of the samples at 450 nm. Hela cells were severed as positive control.

### Statistical Analyses

Data are presented as mean ± S.D. Student’s *t*-test was performed to analyze the differences between two groups. For more than three groups, one-way ANOVA was used, followed by Tukey’s post-test. All data were analyzed and presented by GraphPad Prism 6. The threshold for statistical significance was set at *P* < 0.05.

## Results

### TPP1 Protects OMSCs Against Apoptosis *in vitro*

**FIGURE 1:**
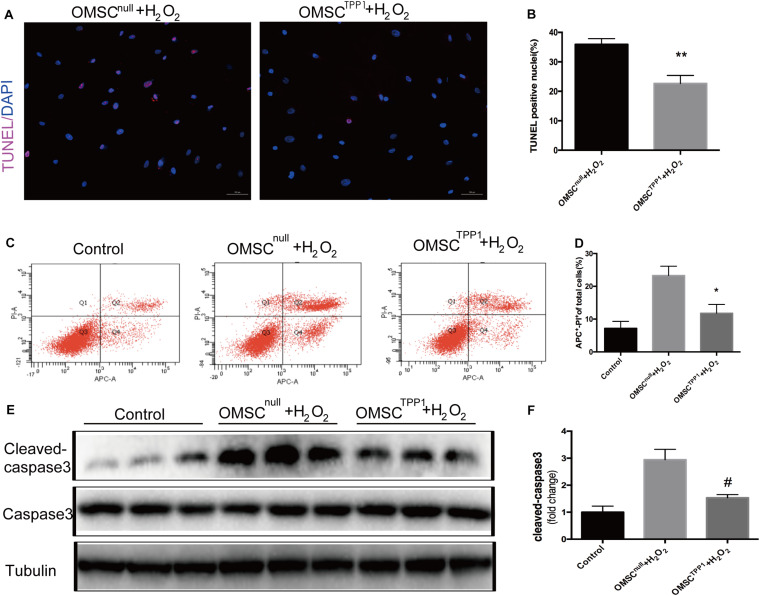
TPP1 protects OMSC from apoptosis *in vitro*. **(A,B)** Representative images of TUNEL staining; the percentages of apoptotic cells were calculated as follows: (TUNEL-positive cells/total nuclei counted)*100%. Five or six fields were randomly selected from the well (at least five wells) in each group to count the TUNEL-positive cells. **(C,D)** Annexin APC/PI staining was analyzed for cell apoptosis and the apoptotic rate of quantification was measured. **(E,F)** The protein level of apoptotic marker cleaved caspase-3 was measured using western blotting. An independent *in vitro* experiment was repeated three times. Data are shown as mean ± S.D. *P < 0.05, ^#^*P* < 0.05, **P < 0.01.

In our previous study, TPP1 expression was found to be upregulated in SIRT1-overexpressing OMSCs, which significantly enhanced the ability of these cells to survive after injection following myocardial infarction (Chen et al., 2014). Thus, we considered that TPP1 likely plays a vital anti-apoptotic role. We found that the level of TPP1 expression was significantly decreased in OMSCs compared to young MSCs (Supplementary Figure 2A); therefore, we chose to overexpress TPP1 in OMSCs (Supplementary Figure 2B). To mimic the microenvironment of myocardial infarction, apoptosis was induced in the OMSCs with 1,000 μM H_2_O_2_ for 2 h. Overexpression of TPP1 significantly attenuated apoptosis of OMSCs, as seen in the reduced apoptotic rate indicated by TUNEL (Figures 1A,B) and Annexin APC/PI assay (Figures 1C,D). Overexpression of TPP1 also significantly decreased cleaved caspase-3 expression through Western blotting (Figures 1E,F). These results demonstrate that TPP1 plays a vital role in protecting OMSCs against apoptosis.

### TPP1 Overexpressing Aged MSC (OMSC^TPP1^) Transplantation Results in Better Recovery of Cardiac Function

**FIGURE 2:**
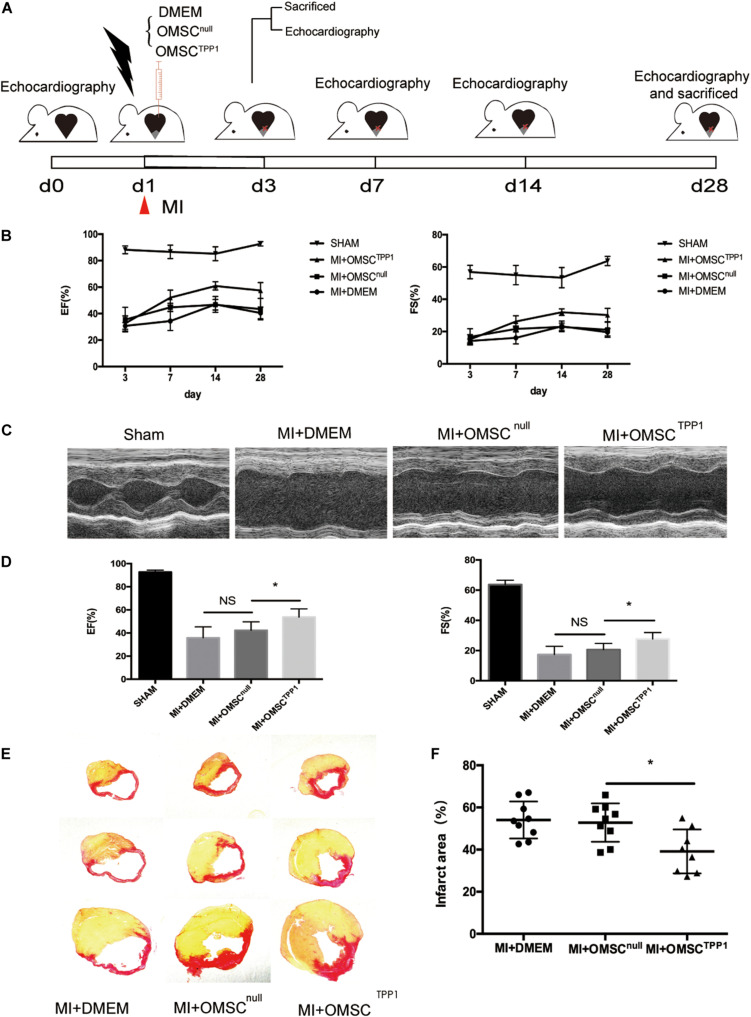
OMSC^TPP1^ improves cardiac functional recovery and reduced cardiac fibrosis after MI. **(A)** Schematic representation of 28-day study of a myocardial infarction mouse model. Echocardiography was performed at days 3, 7, 14, and 28. Animals (*n* = 4–8 per group) were euthanized for tissue harvesting at days 3 and 28. Sham-operated mice served as controls. **(B)** Cardiac function, including ejection fraction (EF) and fraction shortening (FS), at days 3, 7, 14, and 28 after MI. **(C)** Representative echocardiographic images at day 28 after MI. **(D)** EF and FS at day 28 after MI. **(E,F)** The infarct area was determined by Sirius red staining (*n* = 4–8). All data are expressed as mean ± S.D. *P < 0.05.

Next, we employed an MI model in mice(Figure 2A) to determine whether TPP1 overexpression in OMSCs results in better cardiac function. Myocardial contractile parameters were measured at 3, 7, 14, and 28 days after MI (Figure 2B and Supplementary Figures 4E,F). An echocardiographic image at day 28 (Figure 2C) revealed that the transplantation of OMSC^TPP1^ significantly improved systolic function with higher EF and FS than the OMSC^null^ and DMEM groups (Figure 2D). The results from Sirius red staining showed a reduction in infarct scarring (Figures 2E,F). Together, these results confirmed that transplantation of OMSC^TPP1^ could produce a significantly smaller infarct area and better cardiac function than the OMSC^null^ group.

### Enhancing Aged MSC Survival Through TPP1 Overexpression Results in Cardiac Cell Protection and Angiogenesis in Ischemic Hearts

**FIGURE 3:**
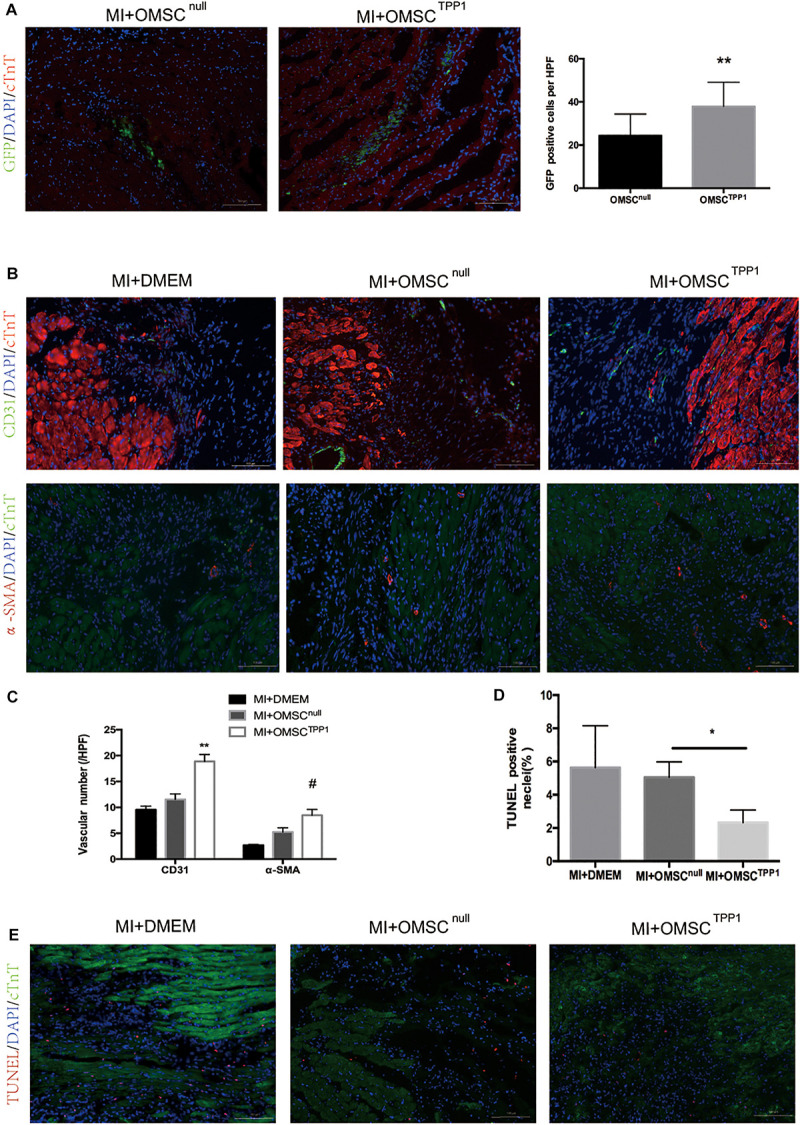
Enhanced OMSC survival after TPP1 overexpression leads to reduced cardiomyocyte loss and enhanced angiogenesis after MI *in vivo*. **(A)** Representative images showing cell survival of GFP-OMSC^null^ and GFP-OMSC^TPP1^ at day 3 post cell transplantation. GFP-positive cells were counted as surviving cells in the infarcted area, *n* = 15–20 from five mice in each group. **(B,C)** Representative immunofluorescence of α-SMA, and CD31 in the infarct border zone of ischemic hearts at day 28 post MI. Angiogenesis was identified by 5–6 high-power fields (HPFs) for each mouse (*n* = 5–6 for each group). **(D,E)** Representative images of a TUNEL staining assay in infarct border zone 3 days after MI; TUNEL-positive cardiomyocytes were quantitatively measured. At least five fields for each mouse were selected to count in each group, *n* = 5–6 mice. Data are shown as mean ± S.D. *P < 0.05, ^#^*P* < 0.05, **P < 0.01.

The efficacy of aged MSC therapy is thought to be limited by the exceptionally small number of transplanted cells remaining in the target tissue. To address this question, we investigated whether TPP1 overexpression could improve OMSC survival *in vivo*. OMSCs were labeled with GFP for transplantation. The results indicated that the OMSC^TPP1^ transplantation group had a higher survival rate than the OMSC^null^ group (Figure 3A).

To assess whether enhanced aged MSC survival mediated by TPP1 could result in greater angiogenesis and less cardiomyocyte death, we detected the density of microvessels and cardiomyocyte apoptosis in the peri-infarct zones by CD31, smooth muscle actin (SMA), and TUNEL staining, respectively. CD31- and SMA-positive vessels were significantly more common in the OMSC^TPP1^ group than in the OMSC^null^ group (Figures 3B,C). TUNEL staining was conducted to detect the cardioprotective effects of OMSC^TPP1^ therapy *in vivo*. The numbers of apoptotic cells in the peri-infarct zone were significantly decreased in OMSC^TPP1^ group compared to the OMSC^null^ and DMEM group (Figures 3D,E).

Furthermore, to investigate the angiogenesis effects of TPP1-modulated OMSCs *in vitro*, we conducted a tube formation assay of HUVECs, which revealed significantly better tube information in the OMSC^TPP1^ and OGD medium group than in the OMSC^null^ and OGD medium group, and there were no statistical differences between the OMSC^TPP1^ and normoxia medium and OMSC^null^ and normoxia medium groups (Supplementary Figures 4A,B). We also evaluated the effects of hMSCs on cardiomyocyte apoptosis *in vitro*. The results demonstrate that less apoptotic cardiomyocytes were found in the OMSC^TPP1^ and OGD medium group than in the OMSC^null^ and OGD medium group (Supplementary Figures 4C,D).

These data indicate that prolonged OMSC survival in oxidative stress results in stronger angiogenesis and cardiomyocyte protection, together with improved myocardial function.

### TPP1 Promotes DNA Repair via MRE11 Without Affecting Telomerase and Other Shelterin Components

Telomerase activity and shelterin components play a pivotal role in telomere length, telomere stability, and cell aging, particularly in stem cells (Blasco, 2007; Rossi et al., 2007). Numerous studies have found that telomerase and shelterin components have a positive impact on cell apoptosis (Arish et al., 2015; Wang et al., 2019). We checked whether TPP1 overexpression affects other shelterin components and found that the transduction of TPP1 did not affect the expression level of other shelterin genes (Supplementary Figures 3A,B). TPP1 overexpression also did not influence telomerase activity (Supplementary Figures 3C,D).

**FIGURE 4:**
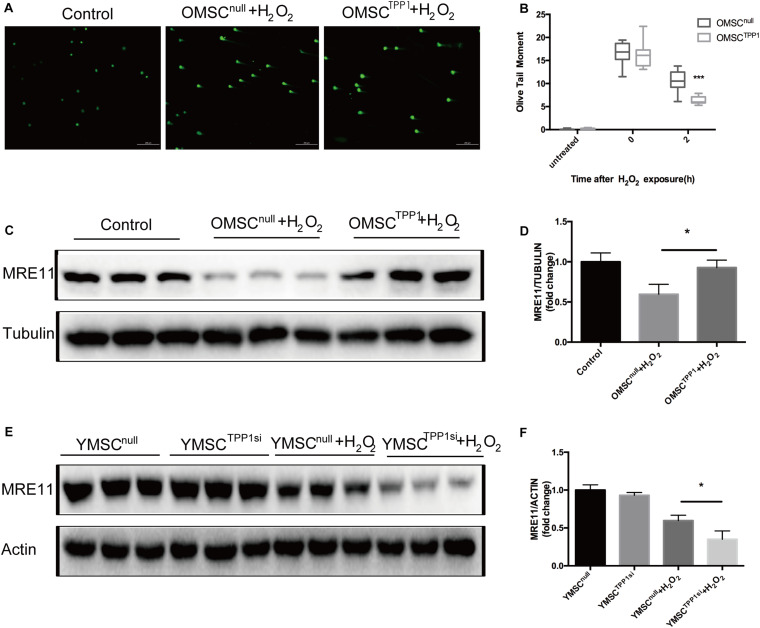
TPP1 enhances DSB repair and upregulates MRE11. **(A)** Alkaline comet assay of OMSCs with vector or TPP1 overexpressed, followed by treatment with 1,000 μmol H_2_O_2_. Cells were treated for 1 h, followed by recovery after 1 h. Comets images were randomly found under microscope on each slide. The average olive tail moment was analyzed (50 cells/slide) using Comet Score Freeware. **(B)** Quantification of the alkaline comet assay olive tail moment. **(C,D)** MRE11 protein level was quantified by western blotting after OMSCs were treated with H_2_O_2_ for the indicated duration, using tubulin as a loading control. **(E,F)** Western blots and quantification protein expression of MRE11 in YMSC^null^ (YMSC transfected with null-siRNA) or YMSC^TPP1si^ (YMSC transfected with TPP1-siRNA). Each experiment was repeated three times. Data are expressed as mean ± S.D. *P < 0.05, ***P < 0.001.

A recent study demonstrated the key role of TPP1 in telomere function and the DNA damage response (Qiang et al., 2014). To examine whether TPP1 is involved in DNA repair, γ-H2AX, which usually appears soon after DNA damage and demarcates the sites of unrepaired double-stranded breaks, was used to evaluate the effects of TPP1 on OMSCs after H_2_O_2_ stimulation. After 1 h H_2_O_2_ treatment, cells were able to repair oxidative damage for 2 h, and the remaining foci were detected by γ-H2AX immunofluorescence. Compared to OMSC^null^, γ-H2AX foci were remarkably decreased in cells transfected with TPP1 after 2 h recovery (Figures 4A,B). MRE11 plays a vital role in DNA repair. Thus, we detected its expression in OMSCs that overexpress TPP1. A significant increase in the protein level of MRE11 was found in OMSC^TPP1^ relative to OMSC^null^ (Figures 4C,D). In addition, MRE11 was downregulated when TPP1 was knocked down by TPP1 siRNA (Supplementary Figure 2C) in young MSCs (YMSCs) (Figures 4E,F).

### TPP1 Promotes DNA Repair Through Activation of AKT Phosphorylation in OMSCs

**FIGURE 5:**
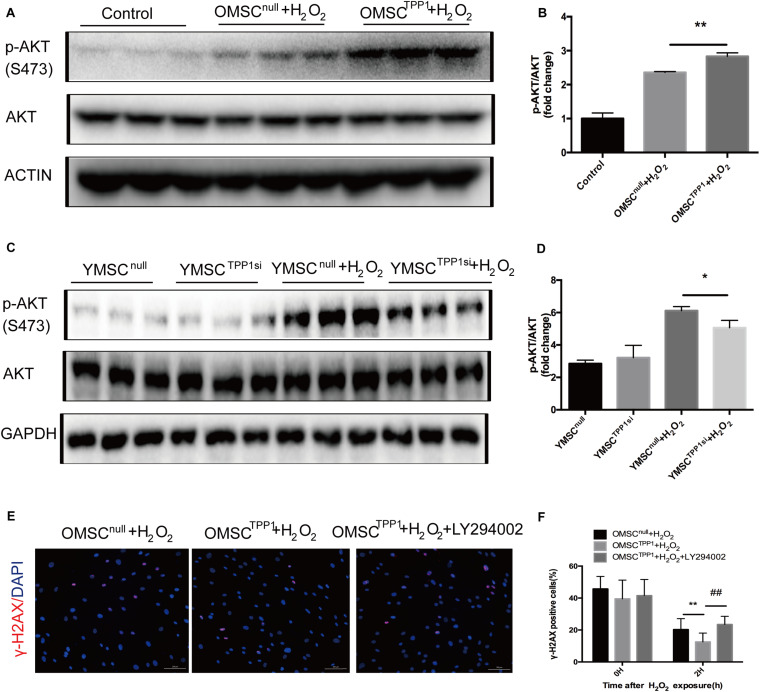
TPP1 enhances DSB repair by AKT activation. **(A,B)** Western blots and quantification of p-AKT (s473) protein expression in OMSCs. **(C)** Western blots and quantification of p-AKT(s473) protein expression in YMSCs. **(D)** Western blots and quantification of p-AKT (s473) and TPP1 protein expression in OMSCs. **(E)** After transfection with lentivirus 24 h, OMSCs were pretreated with DMSO or LY294002 for 24 h and then treated with H_2_O_2_ for 1 h with another 1 h recovery. After this, the cells were fixed and stained for γ-H2AX and nuclei (DAPI) through immunofluorescence. Representative fluorescence graphs show the accumulation of γ-H2AX in cells, magnification 100×. γ-H2AX (red)-positive cells were counted as unrepaired cells through 5–6 high-power fields (HPFs) per well, *n* = 3 well in each group. **(F)** Quantification of γ-H2AX-positive cells in OMSCs after H_2_O_2_ treatment at the indicated times. Data are expressed as mean ± S.D. *P < 0.05, **P < 0.01, ^##^*P* < 0.01.

AKT might augment telomere protection by promoting homodimerization of TPP1 (Han et al., 2013). Therefore, we speculated that TPP1 may induce AKT phosphorylation. As expected, TPP1 enhanced AKT phosphorylation in the presence of H_2_O_2_ in OMSCs (Figures 5A,B), and a decrease in AKT phosphorylation was observed after H_2_O_2_ exposure with TPP1 depletion by TPP1 siRNA (Figures 5C,D). Moreover, a DNA repair assay showed that DNA repair capacity of OMSC^TPP1^ was arrested by LY294002 (PI3K inhibitor), proving that TPP1 facilitates DSB repair through AKT activation (Figures 5E,F).

### MRE11 Is Downstream of AKT and Responsible for TPP1-Induced DNA Repair

**FIGURE 6:**
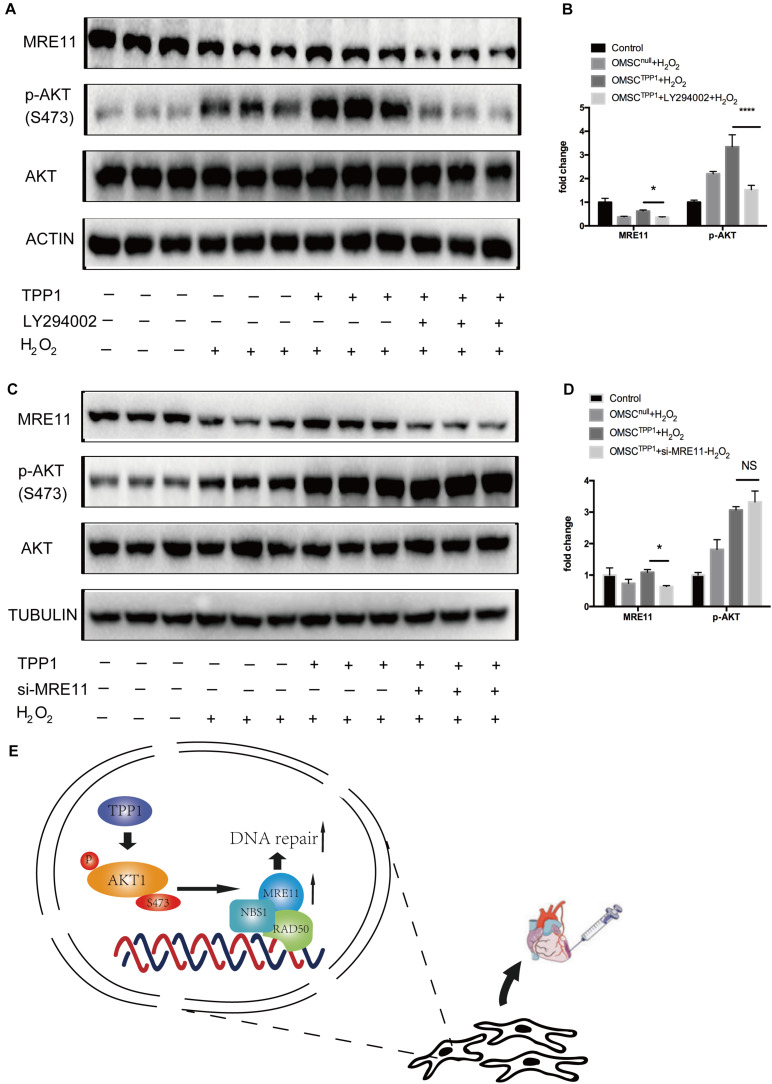
The AKT/MRE11 pathway is responsible for the protective effects of TPP1 in response to oxidative stress. **(A,B)** OMSC^null^ and OMSC^TPP1^ were treated with DMSO or LY294002 for 24 h and treated with H_2_O_2_. Then, the cells were pretreated and lysed, and the levels of the p-AKT, AKT, and MRE11 proteins were detected by western blotting. Actin served as the loading control. **(C,D)** OMSC^null^ and OMSC^TPP1^ were transiently transfected with siRNA (control) or MRE11-siRNA for 48 h and then exposed to H_2_O_2_. The Western blots and quantification for MRE11, p-AKT, and AKT were analyzed. Each experiment was repeated three times. The data are represented as mean ± S.D. *P < 0.05, ****P < 0.0001. **(E)** Schematic diagram showing that TPP1 elevated DSB repair through the AKT-MRE11 pathway to enhance the therapeutic effects of OMSCs transplantation in MI.

To further evaluate the role of MRE11 as the mediator of the effects of TPP1 on DNA repair, we investigated the interaction between AKT and MRE11. Previous studies have found that AKT promotes DNA repair in cancer cells by upregulating MRE11 expression following exposure to ionizing radiation (Deng et al., 2011). We wondered whether the upregulation of MRE11 by TPP1 was mediated by the AKT pathway. Interestingly, MRE11 was downregulated by the PI3K inhibitor LY294002 even after TPP1 overexpression (Figures 6A,B), which means that AKT phosphorylation is necessary for TPP1-induced MRE11 upregulation. To confirm whether MRE11 affects AKT phosphorylation, we transfected siRNA against MRE11 into TPP1-overexpressed OMSCs. The results indicated that MRE11 knockdown did not influence AKT phosphorylation (Figures 6C,D). These findings suggest that TPP1 activates AKT phosphorylation upstream of MRE11 to mediate DNA repair, thereby inducing greater therapeutic effects of OMSCs after their transplantation in MI (Figure 6E).

## Discussion

Mesenchymal stem cell-based cell therapy for ischemic heart disease has made great advances in recent decades. However, improving the therapeutic effects of OMSCs remains a major challenge. In our study, gene expression analyses of YMSCs versus OMSCs revealed that TPP1 was likely to be involved in the process of aging (Supplementary Figure 2A). Numerous studies have found that the levels of shelterin proteins that directly interact with TPP1, such as TRF1 and POT1, are decreased in aging (Derevyanko et al., 2017; Hosokawa et al., 2017). In the present study, the key role of TPP1 in enhancing therapeutic properties of OMSCs was demonstrated. We treated MSCs with H_2_O_2_ to induce oxidative stress to mimic the microenvironment of myocardial infarction, and we confirmed the importance of TPP1 in protecting OMSCs from H_2_O_2_-induced apoptosis. This led to enhanced MSC survival following stresses associated with ischemia, with potential implications for cell therapy in myocardial infarction. Furthermore, our results support a pathway wherein TPP1 activates AKT phosphorylation and then upregulates MRE11 to mediate DNA repair and enhanced the therapeutic properties of OMSCs.

The repair capacity of DSBs usually declines with aging (Pan et al., 2016) and is associated with a decreased expression of DNA-repair proteins binding with the telomere (Ju et al., 2006). A previous study revealed that TPP1 blocks DSB resection, mediated by ATR (Kibe et al., 2016). MRE11, an important component of DNA repair, is critical for DNA end resection and regulating DSB repair. It is also a key part of the MRN complex, which is involved in non-homologous end-joining (NHEJ) and homology-directed repair (Stracker and Petrini, 2011). In addition, MRE11 had frequently been found to decrease in aged humans and other animals, with reduced NHEJ activity as well (Ju et al., 2006; Vyjayanti and Rao, 2006). It has also been found that MRE11 downregulation impairs the repair capacity of radiation-induced DSB in cancer cells (Nicholson et al., 2017). In addition, MRE11 interacts with TRF2 to play a protective role in telomere fusion after replication (de Lange and Petrini, 2000; Deng et al., 2009). Here, we found that TPP1 could maintain MRE11 stabilization under oxidative stress and is the critical factor in the process of TPP1 enhancement of DSB repair and cell anti-oxidative stress capacity in human MSCs.

Many cellular processes can be attributed to AKT signaling, including apoptosis, proliferation, migration, and cell cycle regulation (Manning and Toker, 2017). In addition, several studies have demonstrated that AKT activity is involved in telomere protection and DNA repair (Han et al., 2013; Liu et al., 2014). AKT inhibition has also been shown to disrupt TPP1 and POT1 recruitment to the telomere (Han et al., 2013). Here, we revealed non-telomeric mechanisms of AKT, which are partly in accordance with a previous study that found that AKT interacts with TPP1 and regulates TPP1 homodimerization (Han et al., 2013). Our data showed that TPP1 activates AKT, which consequently upregulates MRE11 to enhance DSB repair. It is remarkable that the AKT-MRE11 pathway in OMSCs with overexpressed TPP1 was not activated in the absence of oxidative stress (Supplementary Figure 2D). Based on this molecular mechanism, it is reasonable to explain the *in vitro* results of tube formation, such that no difference was found between the OMSC^TPP1^ and normoxia medium group and the OMSC^null^ and normoxia medium group.

Taken together, our results highlight the non-telomeric mechanisms by which TPP1 regulates aged MSC function and DSB repair capacity. We provide evidence that the mechanism involves the mutual regulation between TPP1 and AKT, and activation of MRE11 to enhance DNA repair. This work provides new mechanistic insight into autologous MSC transplantation therapy in aged individuals and indicates TPP1’s role as a key element promoting DNA repair in the AKT/MRE11 pathway.

## Data Availability Statement

The raw data supporting the conclusions of this article will be made available by the authors, without undue reservation, to any qualified researcher.

## Ethics Statement

The studies involving human participants were reviewed and approved by the Ethics Committee of the Second Affiliated Hospital of Zhejiang University. The patients/participants provided their written informed consent to participate in this study. The animal study was reviewed and approved by the animal care and use Committee of Zhejiang University.

## Author Contributions

KY and ZZ designed the study. SC, WH, CG, and FL performed the analyses and produced the initial draft of the manuscript. JC, YQ, DX, and JZ contributed to the drafting of the manuscript. XL and JW provided conception and design, administrative support, financial support, and final approval of the manuscript. All authors have read and approved the final submitted manuscript.

## Conflict of Interest

The authors declare that the research was conducted in the absence of any commercial or financial relationships that could be construed as a potential conflict of interest.

## Abbreviations

MSC: mesenchymal stem cell
OMSC: aged MSC
OMSC^null^: OMSC infected with Len-null
OMSC^TPP1^: OMSC infected with Len-TPP1
OGD: oxygen glucose deprivation
YMSC: young MSCs
YMSC^null^: YMSC transfected with null-siRNA
YMSC^TPP1si^: YMSC transfected with TPP1-siRNA.
