# Supplementary material for: TPP1 Enhances the Therapeutic Effects of Transplanted Aged Mesenchymal Stem Cells in Infarcted Hearts via the MRE11/AKT Pathway
Source: Front Cell Dev Biol. 2020 Oct 29;8:588023. doi: 10.3389/fcell.2020.588023 (PMC7658181; doi:10.3389/fcell.2020.588023)
Supplement: Supplementary file 1 [file Image_1.pdf]

Supplementary Material

Supplementary Figure.1

A-E. Flow cytometric examination shows that hMSCs were positive for CD29,CD44 and CD90,and negative for CD34 and CD45. F-G. Adipogenesis, osteogenesis and chondrogenesis differentiation of hMSCs were conducted by oil red O staining (F),alizarin red staining (G) and toluidine blue staining (H), respectively.

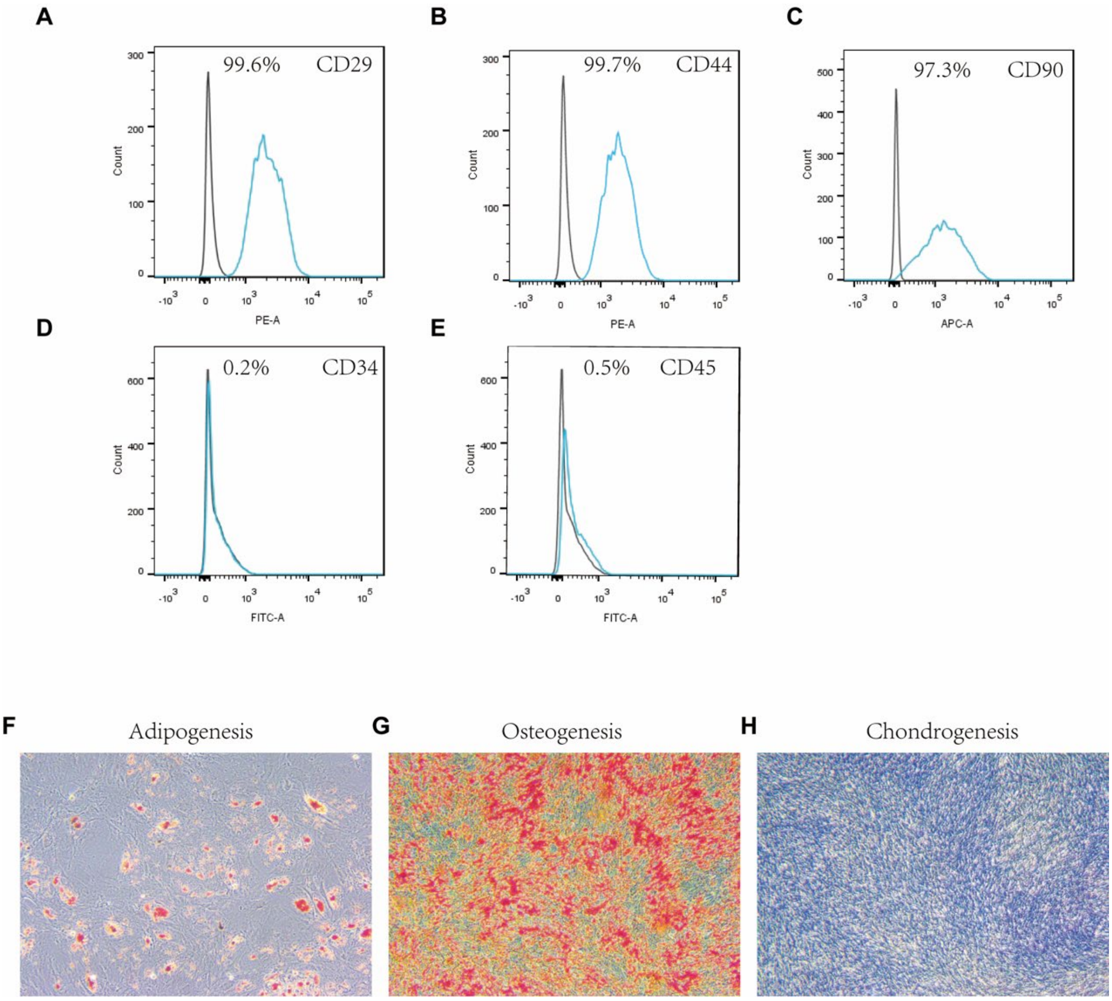

Supplementary Figure.2

A.TPP1 expression between YMSCs and OMSCs B.TPP1-flag lentivirus overexpressed TPP1 expression in OMSCs. C.TPP1 siRNA decreased TPP1expression in YMSCs.

D.MRE11 and p-AKT expression of OMSCs in the normal condition.Data were shown as mean ± S.D.* $P < 0.05$. **$P < 0.01$. ****$P < 0.0001$.

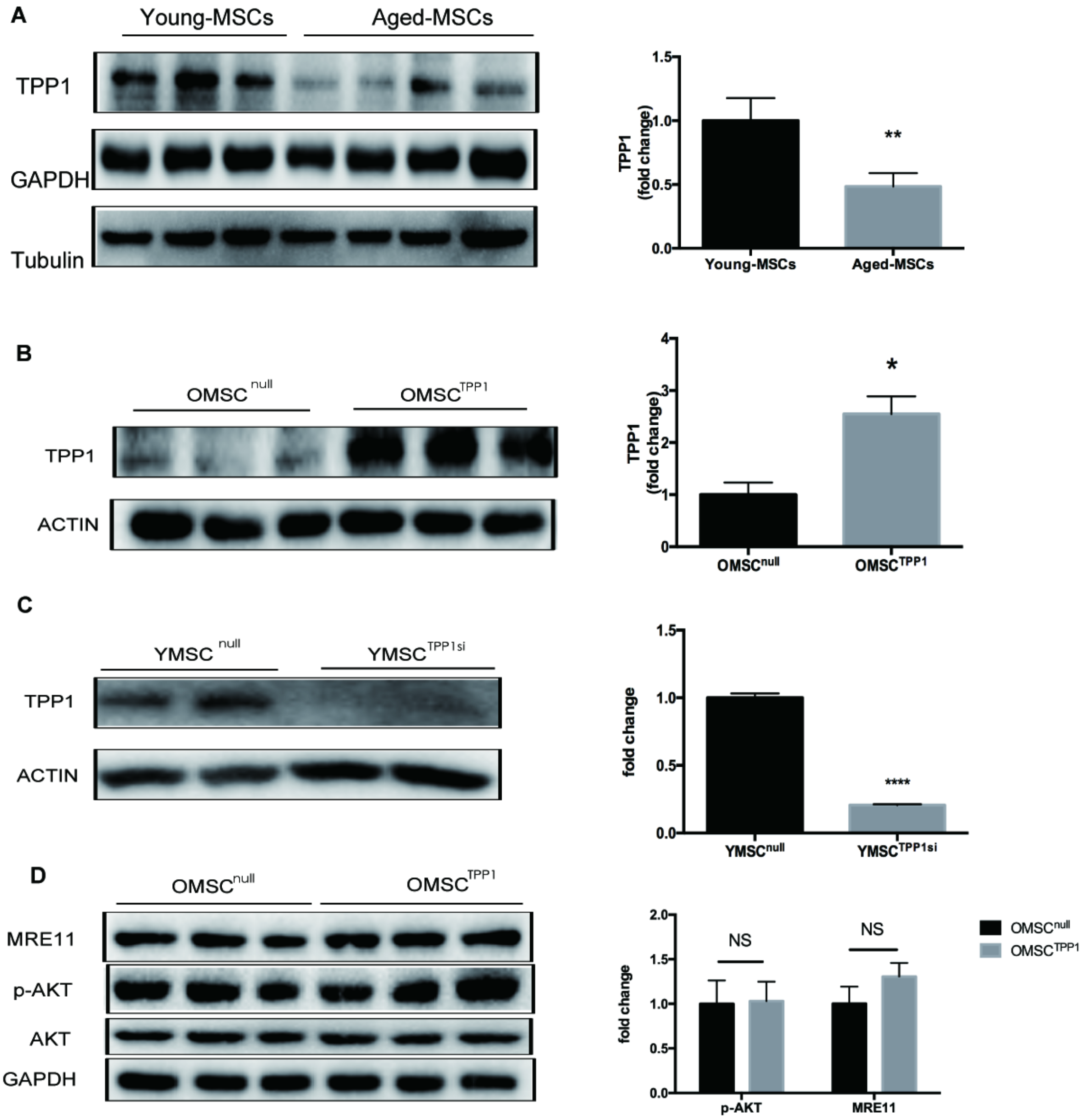

Supplementary Figure.3

A-D Shelterin components and telomerase analysis of (A) POT1 and TIN2 protein expression (B) Quantification RNA expression of POT1,TIN2,TRF2,TRF1 and RAP1 (C)Quantification RNA expression of TERT (D) telomerase activity in TPP1 overexpressed OMSCs. Data were shown as mean ± S.D.

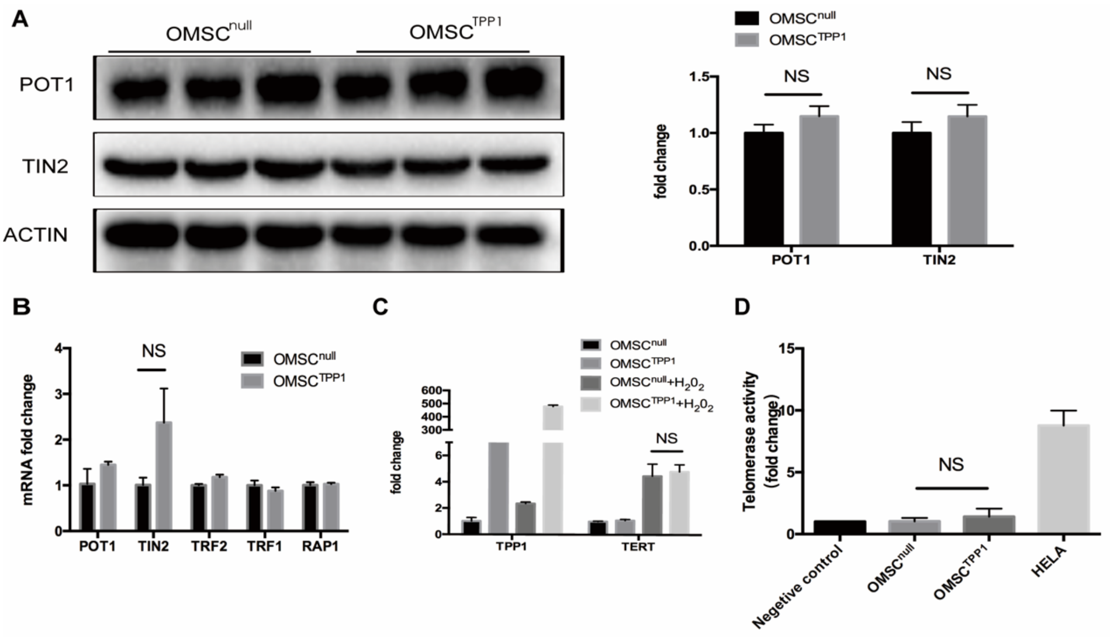

Supplementary Figure.4

A.Tube formation was performed using GFP-HUVECs cultured with conditioned medium collected from DMEM alone, OMSC^{null} normal cultured DMEM,OMSC^{TPP1} normal cultured DMEM, OMSC^{null} OGD cultured DMEM and OMSC^{TPP1} OGD cultured DMEM. The conditioned medium had been normalized by 1×10^6 number of hMSCs. B.The quantification of tube formation in each group. n=3-6 wells in each group.C.D.The quantification and image of the apoptotic rate of H9C2 cardiomyocytes measured by Annexin APC/PI staining . E.Representative echocardiographic images at 7 days and 14 days after MI. F.Ejection fraction (EF) and Fraction shortening (FS), at days 7 and 14 after MI. Data were expressed as mean ± S.D.* $P < 0.05$. **$P < 0.01$, ***$P < 0.001$

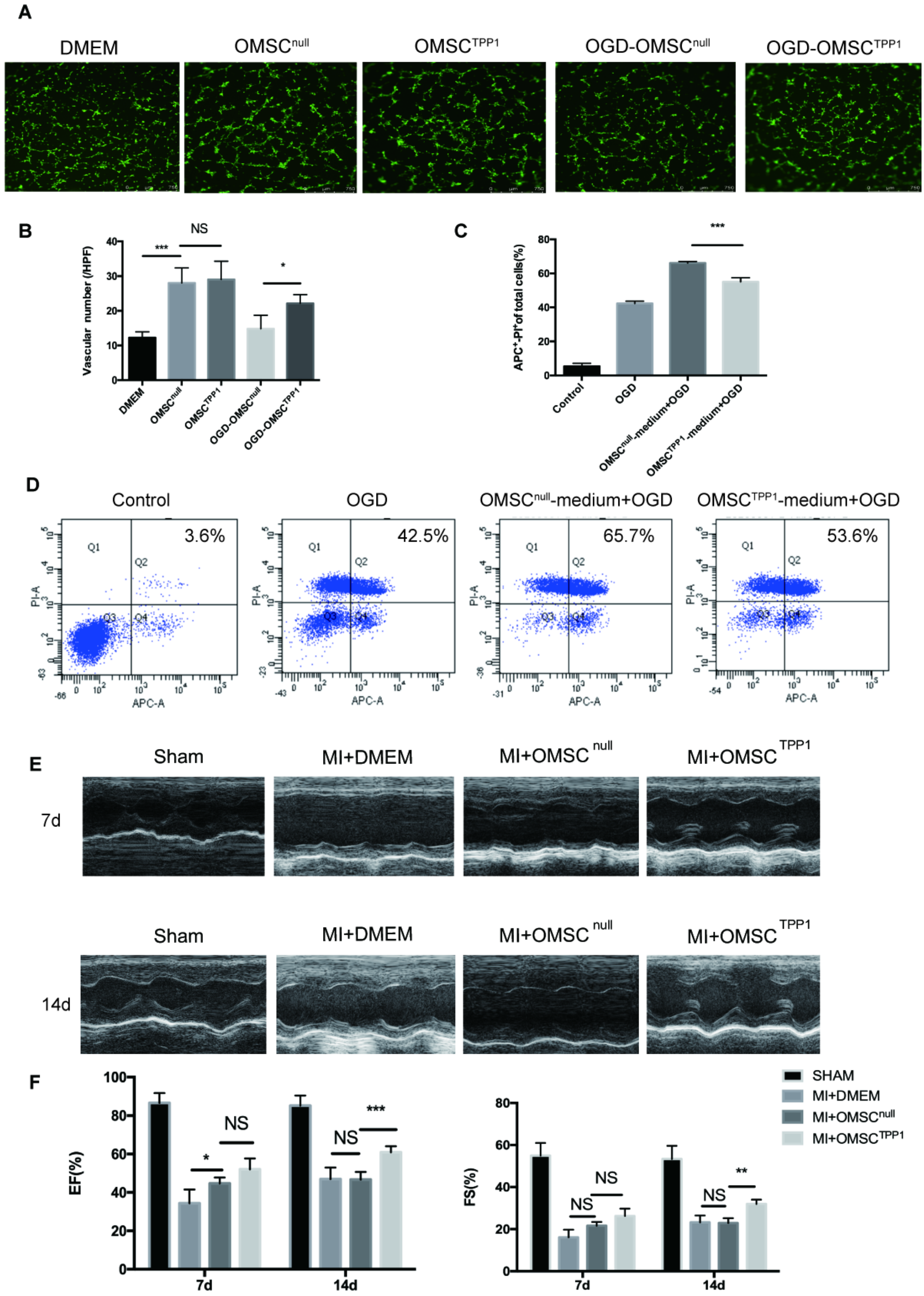

A
DMEM
OMSCnull
OMSCTPP1
OGD-OMSCnull
OGD-OMSCTPP1
B
Vascular number (/HPF)
NS
***
*
C
APC+-PI+of total cells(%)
***
Control
OGD
OMSCnull-medium+OGD
OMSCTPP1-medium+OGD
D
Control
OGD
OMSCnull-medium+OGD
OMSCTPP1-medium+OGD
3.6%
42.5%
65.7%
53.6%
Q1
Q2
Q3
Q4
PI-A
APC-A
E
Sham
MI+DMEM
MI+OMSC null
MI+OMSC TPP1
7d
14d
F
EF(%)
FS(%)
7d
14d
NS
*
***
**
SHAM
MI+DMEM
MI+OMSCnull
MI+OMSCTPP1

Supplementary Table1 Primer list

hum TPP1 F	CCTGCTCTGTCTGGTATGAGT
hum TPP1 R	GAGTGTGGAGCGGTATCTGT
hum ACTIN F	CCCTGGAGAAGAGCTACGAG
hum ACTIN R	CGTACAGGTCTTTGCGGATG
hum TERT F	CCTTCCGCCAGGTGTCC
hum TERT R	GGAGCCACCAGCACAAAGA
hum POT1 F	CGGCAGCAAGTTGTCAGATT
hum POT1 R	CTGGCTGTAGGGATCCGAAA
hum TRF2 F	AGCCACTTGTGACTCCATGA
hum TRF2 R	TAGCCCAGCAGATGTTGACA
hum TRF1 F	GGCTGACCTCAACCCTGTAA
hum TRF1 R	AAAATTAGCTGGGCGTGGTG
hum TIN2 F	CTGTGGATTTGGCCTCGAAG
hum TIN2 R	CCAGGCAAGAGAAGAGGTGA
hum RAP1 F	CCGCTAACATTGCTTCGACA
hum RAP1 R	TCTAGCACTTCACCACCGTT
